# *In vitro* characterization of the phage lysis protein MS2-L

**DOI:** 10.20517/mrr.2023.28

**Published:** 2023-07-20

**Authors:** Julija Mezhyrova, Janosch Martin, Clara Börnsen, Volker Dötsch, Achilleas Stefanos Frangakis, Nina Morgner, Frank Bernhard

**Affiliations:** ^1^Institute of Biophysical Chemistry and Center for Biomolecular Magnetic Resonance, Goethe University, Frankfurt am Main 60438, Germany.; ^2^Institute of Physical and Theoretical Chemistry, Goethe University, Frankfurt am Main 60438, Germany.; ^3^Buchmann Institute for Molecular Life Sciences & Institute of Biophysics, Goethe University, Frankfurt am Main 60438, Germany.

**Keywords:** Phage toxins, cell-free expression, native mass spectrometry, molecular assemblies, nanodiscs

## Abstract

**Background:** The peptide MS2-L represents toxins of the ssRNA Leviviridae phage family and consists of a predicted N-terminal soluble domain followed by a transmembrane domain. MS2-L mediates bacterial cell lysis through the formation of large lesions in the cell envelope, but further details of this mechanism as a prerequisite for applied bioengineering studies are lacking. The chaperone DnaJ is proposed to modulate MS2-L activity, whereas other cellular targets of MS2-L are unknown.

**Methods:** Here, we provide a combined *in vitro* and *in vivo* overexpression approach to reveal molecular insights into MS2-L action and its interaction with DnaJ. Full-length MS2-L and truncated derivatives were synthesized cell-free and co-translationally inserted into nanodiscs or solubilized in detergent micelles. By native liquid bead ion desorption mass spectrometry, we demonstrate that MS2-L assembles into high oligomeric states after membrane insertion.

**Results:** Oligomerization is directed by the transmembrane domain and is impaired in detergent environments. Studies with truncated MS2-L derivatives provide evidence that the soluble domain acts as a modulator of oligomer formation. DnaJ strongly interacts with MS2-L in membranes as well as in detergent environments. However, this interaction affects neither the MS2-L membrane insertion efficiency nor its oligomerization in nanodisc membranes. In accordance with the in vitro data, the assembly of MS2-L derivatives into large membrane located clusters was monitored by overexpression of corresponding fusions with fluorescent monitors in *E. coli* cells. Analysis by cryo-electron microscopy indicates that lesion formation is initiated in the outer membrane, followed by disruption of the peptidoglycan layer and disintegration of the inner membrane.

**Conclusion:** MS2-L forms oligomeric complexes similar to the related phage toxin ΦX174-E. The oligomeric interface of both peptides is located within their transmembrane domains. We propose a potential function of the higher-order assembly of small phage toxins in membrane disintegration and cell lysis.

## INTRODUCTION

The 75-amino acid protein MS2-L is a prototypical toxin of the ssRNA *Leviviridae* group of bacteriophages. The toxin shows an amphiphilic topology consisting of a proposed N-terminal soluble domain followed by a transmembrane domain. MS2-L is extremely toxic to bacteria and its recombinant expression rapidly kills *E. coli* cells by forming lesions in the cell envelope and subsequent release of cytoplasmic content^[1,2]^. The essential lytic activity of MS2-L was confined to the C-terminal 35 amino acids containing its transmembrane domain and deletion of the soluble domain did not affect its lytic function^[3]^. The chaperone DnaJ was identified as an interaction partner of MS2-L. Its interaction with the soluble domain of MS2-L was shown in pulldown experiments. Deletion of this domain abolished DnaJ interaction, while lysis function remained unaffected. Thus, the chaperone action seems not to be essential for cell envelope disintegration^[4]^. The presence and nature of further MS2-L targets in the bacterial membrane are still unclear. Previous electron microscopy studies using immunogold staining localized MS2-L in bacterial cell membranes and to a major part within membrane adhesion sites^[5]^. An early postulation was the oligomerization of the MS2-L transmembrane domain^[6]^. Furthermore, lysis as a result of inducing the bacterial autolytic system was discussed^[1,7]^.

MS2-L-like toxins are related to the ΦX174-E toxin family of the ssDNA *Microviridae* bacteriophages. They are designated as “amurins” or “single gene lysis (Sgl)” proteins as they are proposed to inhibit specific steps in bacterial peptidoglycan synthesis^[7-9]^. Both toxins may play major roles in the future development of novel antibiotics and they are of increasing commercial interest due to their ability to produce bacterial ghost cells suitable for vaccination. Additionally, they find applications in structural biology, e.g., as a tool for electron tomography^[10,11]^. ΦX174-E membrane insertion strongly depends on its interaction with the chaperone SlyD and the lipid-I precursor forming enzyme MraY has been identified as one target within the bacterial membrane^[12-17]^. Similar to MS2-L, ΦX174-E consists of one proposed transmembrane domain connected to a short soluble domain. However, the organization of the domains is reversed and the transmembrane domain of ΦX174-E is N-terminally located^[8]^.

Successful phage infection is a complex mechanism depending on physiological conditions, growth phase characteristics and gene expression regulation by the bacterial host cells. Delicate stoichiometry and production timing of individual process compounds can be pivotal for the formation of essential interactions and for a coordinated cycle of phage replication and host lysis. However, the implementation of phage toxins such as MS2-L as biochemical tools in antibiotic development or ghost technology usually requires their overproduction and usage in non-physiological concentrations. The major goal of this study was, therefore, to characterize the function of the MS2-L toxin *in vitro* in a defined cell-free (CF) system and in overproducing bacterial cells in order to provide a basic platform for future applications. In previous work, we demonstrated the formation of ΦX174-E oligomers and the toxin’s co-translational interaction with SlyD by a combined approach including laser-induced liquid-bead ion desorption (LILBID) mass spectrometry, nanodisc (ND) technology and CF expression studies^[17]^. SlyD forms a stable complex with the nascent soluble domain of ΦX174-E and presumably prevents the formation of an intramolecular interaction between the soluble domain and the transmembrane domain that would result in inactive ΦX174-E. The ΦX174-E/SlyD complex stays soluble and keeps the toxin in a membrane insertion competent conformation. After membrane insertion, ΦX174-E polymerizes into high-order complexes that are presumably essential for subsequent lesion formation and cell lysis. Based on the striking similarities of the MS2-L and ΦX174-E lytic pathways, we now have extended our studies to the membrane insertion mechanism of MS2-L and complex formation with its interaction partner DnaJ. The possibility to insert MS2-L derivatives co-translationally into preformed ND membranes in defined CF reaction environments facilitated an evaluation of the effects of supplied DnaJ.

## METHODS

### DNA techniques

The coding regions of MS2-L derivatives (MS2-L, MS2-L-GFP, MS2-Lp_23_, MS2-Lp_26_, MS2-Lp_29_, MS2-Lp_32_, MS2-Lp_35_) and the *dnaJ* gene were cloned into the NdeI and XhoI restriction sites of vector pET21a (Novagen). All peptides were modified with either a C-terminal StrepII-tag or a GFP-His_10_ fusion separated by a GTGG linker. The full-length constructs MS2-L and MS2-L-GFP contained a codon exchange from GAA (Glu) to AAA (Lys) at the first amino acid position following the start codon methionine^[18]^. The first ten codons of MS2-Lp_29_ and MS2-Lp_32_ were optimized for *E. coli* codon usage to improve expression efficiencies. The construct mScarlet-MS2-L was cloned into the vector pET29 (NdeI/XhoI). The construct contains a GGTG linker between mScarlet and MS2-L and a GG linker between MS2-L and a C-terminal StrepII-tag.

### Cell-free expression reaction

All toxin derivatives were synthesized in a two-compartment continuous exchange cell-free (CF) system consisting of a reaction mixture (RM) and a feeding mixture (FM) as described previously^[19]^. As a lysate source, S30 lysates either from strain A19 or from the *dnaJ* deficient strain BW25113ΔDnaJ were used^[20]^. S30 lysate and T7 RNA polymerase were prepared as previously described in detail^[21,22]^. For all CF expression reactions, a defined RM to FM ratio of 1:15 was applied and a previously determined Mg^2+^ optimum of 20 mM was used for toxin expression. The co-translational solubilization of the synthesized hydrophobic peptides was achieved either by adding defined concentrations of preformed NDs [lipid-based cell-free expression (L-CF) mode] or by supplementing the reaction with 0.4% of the detergent Brij78 [detergent-based cell-free expression (D-CF) mode].

### Preparation of nanodiscs

NDs were preformed with the MSP1E3D1 scaffold protein and either 1,2-dimyristoyl-sn-glycero-3-phosphocholine (DMPC) or 1,2-dimyristoyl-sn-glycero-3-phospho-(1’-rac-182 glycerol) (DMPG) lipids. MSP1E3D1 was expressed and purified as previously published^[17,23]^. NDs were formed by combining purified MSP1E3D1 with the selected lipid and 0.1% dodecyl phosphocholine (DPC). The stoichiometry of MSP1E3D1 to lipid was 1:115 for DMPC and 1:110 for DMPG, respectively. The mixture was incubated for 1 h at room temperature (RT) on a shaking device. Detergent removal was further achieved by subsequent dialysis against 3 × 5 L of DF buffer (10 mM Tris-HCl pH 8.0, 100 mM NaCl) at RT. Each dialysis step was carried out for at least 12 h. After dialysis, NDs were centrifuged at 22,000 × *g* and 16 °C for 20 min and concentrated by ultrafiltration (MWCO 10 kDa) to a concentration of 500-1,000 µM.

### DnaJ expression and purification

For overnight starter cultures, 200 mL LB medium supplemented with 100 µg/mL ampicillin was inoculated with *E. coli* T7 Express cells transformed with pET21-dnaJ. 10 L LB medium supplemented with 100 µg/mL ampicillin and 100 mM glucose were then inoculated 1:100 and incubated at 37 ^o^C and 180 rpm. Cells were grown until OD_600_ reached 0.6 and protein expression was induced with the addition of 1 mM isopropyl-β-D-thiogalactopyranoside (IPTG). The cells were further grown for 3 h at 37 °C, harvested by centrifugation (4,500 × *g*, 4 °C, 30 min) and stored at -80 °C until further use. For purification, the cell pellet of a 10 L expression was resuspended in 50 mL DnaJ buffer (50 mM Tris-HCl pH 7.5, 400 mM NaCl, 10% (v/v) glycerol, 1 mM Tris(2-carboxyethyl)phosphine hydrochloride (TCEP), 0.1% Triton X-100) containing 1× complete protease inhibitor cocktail and disrupted by sonication (sonication steps of 3 × 1 min and 3 × 45 s with cooling intervals of 1 min in between each step). After centrifugation (30,000 × *g*, 4 °C, 30 min), the lysate was filtered (0.45 µm) and applied on Ni^2+^-NTA agarose (3 mL beads) pre-equilibrated with 5 column volumes (cv) of DnaJ buffer. Unbound protein was removed by washing with 5 cv of DnaJ buffer and 5 cv of DnaJ buffer containing 50 mM imidazole. DnaJ was finally eluted in 2 cv of DnaJ buffer supplemented with 300 mM imidazole. Protein-containing fractions were concentrated by ultrafiltration (MWCO 10 kDa), flash-frozen in liquid nitrogen and stored at -80 °C until further use. Since DnaJ exhibited sensitivity against lower temperatures, all purification steps were performed at RT.

### Purification of MS2-L derivatives

CF expressed MS2-L derivatives were purified by affinity chromatography via their StrepII-tag. After expression, the RM was centrifuged (14,000 × *g*, 4 °C, 10 min) and the supernatant was diluted 1:3 in Strep buffer (100 mM Tris-HCl pH 8.0, 100 mM NaCl). When samples were expressed in the D-CF mode, the working buffer was supplemented with 0.02 % Brij78. 100 µL StrepII-Tactin resin was used for 150 µL of RM and equilibrated with 5 cv of Strep buffer. The diluted supernatant was applied on the resin and the flow-through was re-applied three times to achieve optimal protein binding. After washing with 5 cv Strep buffer, proteins were eluted with 2 cv Strep buffer containing 15 mM d-desthiobiotin. Samples were subsequently concentrated by ultrafiltration (MWCO 10 kDa) and used for further analysis.

### Tris-Tricine SDS-PAGE and immunoblotting

Precipitate forming cell-free expression (P-CF) expressed proteins were resuspended in an S30 buffer C [10 mM Tris-acetate pH 8.2, 14 mM Mg(OAc)_2_, 60 mM KOAc] in a volume corresponding to the RM volume. All protein samples were supplemented with 1× SDS loading buffer (4×: 100 mM Tris-HCl pH 6.8, 8 M urea, 20% (w/v) SDS, 20% (v/v) β-mercaptoethanol, 15% (v/v) glycerol, 0.12% (w/v) bromphenol blue), incubated at RT for 10 min and separated using denaturing, discontinuous 4%-11% Tris-Tricine SDS-PAGE as described earlier^[24]^. Separation was carried out at 90 V for 10 min and 150 V for another 45 min. Subsequently, the gels were either fixated in fixing solution [50% (v/v) ethanol, 10% (v/v) acetic acid] and stained with colloidal Coomassie Brilliant Blue G250 staining solution [0.02% (w/v) Coomassie Brilliant Blue G-250, 5% (w/v) aluminium sulfate-(14-18)-hydrate, 10% (v/v) ethanol, 2% (v/v) ortho-phosphoric-acid] or used for immunodetection via Western Blotting. Following SDS-PAGE, proteins were transferred onto a methanol activated 0.45 µm PVDF membrane (340 mA, 35 min) in Towbin buffer [25 mM Tris-HCl pH 8.3, 192 mM glycine, 15% (v/v) methanol]. After protein transfer, the membrane was blocked for 1 h at RT in 4% (w/v) skim milk powder in phosphate-buffered saline with Tween 20 (PBS-T) [2.6 mM KCl, 1.8 mM KH_2_PO4, 137 mM NaCl, 10 mM Na_2_HPO_4_, 0.05% (v/v) Tween-20] and incubated with the 1st antibody (α-StrepII-HRP: 1:7,000 dilution in 0.5% skim milk powder in PBS-T or α-His: 1:2,000 dilution) under the same conditions. In the case of the α-His antibody, the membrane was washed three times with PBS-T and incubated with α-mouse-HRP (1:5,000 dilution in 0.5% skim milk powder in PBS-T) for 1 h at RT or at 4 °C overnight. After incubation, the membrane was washed three times with PBS-T and analyzed by chemiluminescence detection.

### Liquid bead ion desorption mass spectrometry

LILBID is a native mass spectrometry ionization technique in which ion release results from irradiating small microdroplets (Ø 30 nm) containing the analyte of interest with an IR laser^[25]^. The microdroplets are generated with a commercially available piezo-driven droplet generator (MD-K-130; Microdrop Technologies GmbH, Norderstedt, Germany). Droplet irradiation occurs at a frequency of 10 Hz and the IR laser operates at the absorption wavelength of water (2.94 µm). The laser energy can be varied between a range of 10 and 23 mJ. Latter enables the study of intact as well as partially dissociated non-covalently bound protein/protein complexes. A homebuilt time-of-flight setup including a reflectron operating at high vacuum conditions (10^6^ mbar) is used as an analyzer. For more details regarding LILBID-MS, please see^[26]^. A solution of 50 mM ammonium acetate (pH 7.4) was used as buffer for LILBID-MS analysis of full-length MS2-L and truncated constructs in NDs. For analysis of detergent-solubilized MS2L derivatives, a buffer of 50 mM ammonium acetate (pH 7.4) supplemented with 0.02% Brij78 was used. Buffer exchange was performed with the help of Zeba Micro Spin desalting columns (Thermo Scientific, USA, 7 kDa MWCO). A sample volume of 4 µL was used for each measurement. Ion detection was performed in negative ion mode and all shown mass spectra are normalized to 1 and represent averaged signals of 1,000 droplets. *Massign* was used for spectra and data processing^[27]^.

### DnaJ pulldown

Each pulldown reaction contained 60 µL of a 5% magnetic bead suspension (MagStrep “type3” XT beads, iba Lifesciences). The beads were washed 3 times with 1 mL ddH2O and 3× with 1 mL StrepII buffer. If detergent-solubilized proteins were utilized, the working buffer was supplemented with 0.02% Brij78. 50 µg of purified, StrepII-tagged bait proteins (MS2-L derivatives) were immobilized by incubating them with the beads in StrepII buffer (total volume 500 µL) for 1 h at 4 °C in an overhead rotor device. To remove unbound bait, the beads were washed 3× with 1 mL of Strep buffer. Subsequently, a defined amount of the prey protein DnaJ (50 µg) was added and incubated for 4 h at 4 °C in an overhead rotor device for binding. Finally, unbound prey was removed by washing 3× with 1 mL of Strep buffer and the protein complexes were eluted by adding 40 µL of SDS loading buffer and heating at 75 °C for 10 min.

### *In vivo* expression of MS2-L derivatives

Plasmids containing toxin constructs were transformed into Lemo21 (DE3) or T7 Express cells. The transformed cells were plated on LB-agar plates containing the appropriate antibiotic and incubated overnight at 37 °C. To minimize the accumulation of escape mutations against toxins, which could possibly be synthesized by background expression, bacterial cultures (200 mL LB medium with the appropriate antibiotic or a combination thereof) were inoculated directly from the agar plates the next morning. The cultures were incubated at 37 °C and 180 rpm until an OD_600_ of 0.2-0.3 and toxin synthesis was then induced with 1 mM IPTG. Bacterial growth was monitored by periodic OD_600_ measurements. For subsequent LSM imaging, 2 mL bacterial samples were taken at the indicated time points, centrifuged (5,000 × *g*, 4 °C, 2 min), resuspended in 100 µL of 10 mM Tris-HCl, pH 8.0, and flash frozen in liquid nitrogen. Samples were stored at -80 °C until further use.

### Confocal laser scanning microscopy

To capture living bacterial cells, agarose pads (1% agarose in 89 mM Tris-borate, 2 mM EDTA, pH 8.0) were prepared and arranged on glass slides. For bacterial viability assays, the LIVE/DEAD^TM^ BacLight^TM^ Bacterial Viability Kit (ThermoFisher Scientific) was used. A dilution of the propidium iodide (PI) dye (0.3 µL PI + 100 µL PBS) was prepared and 0.5 µL of it was added to 15 µL of the thawed bacterial culture. Cells were then incubated for 15 min at RT under light exclusion. Subsequently, the cells were centrifuged (14,000 × *g*, RT, 2 min), the supernatant was discarded and the cells were washed once with 100 µL PBS. After another centrifugation step at the previously described conditions, cells were resuspended in 15 µL PBS. 3 μL of either stained or directly thawed bacterial culture was pipetted on the agarose pad and a cover slip was placed on top of the pad and fixated with fimo clay. Confocal fluorescence images were acquired with a laser scanning microscope (Zeiss LSM 700) using a Plan-Apochromat 63 × 1.40 Oil DIC objective and Zeiss LSM software. LED lasers [488 nm for green fluorescent protein (GFP), 555 nm for mScarlet and PI] were used at 0.2%-4% intensity and the signal was captured with a PMT detector using a SP555 or LP560 filter, respectively. Gain settings were individually adjusted for each sample, ranging between 500 and 900. The pinhole was set to 1 airy unit (45 μm) and images were acquired with a pixel size of 99 nm and a pixel dwell time of 1.58 μs. To maximize the signal-to-noise ratio, averaging of two scans was performed line by line. All images were processed with the ImageJ software.

### Cryo-electron microscopy sample preparation and data collection

MS2-L toxin expression in *E. coli* Lemo21 (DE3) cells was induced with 1 mM IPTG at OD_600_ = 0.27. 2 mL of cells were taken 60 min after induction, harvested, and resuspended in 250 µL LB-medium. 3.5 µL of sample was deposited onto glow-discharged Quantifoil R3.5/1, 200-mesh Cu holey carbon coated grids and vitrified in liquid ethane using a Vitrobot (FEI, Eindhoven, Netherlands) at 100% humidity and 4 °C. Data was collected with a Titan Krios transmission electron microscope (FEI, Eindhoven, Netherlands) operating at 300  kV with a post-column energy filter in zero-loss peak mode (Gatan Inc., GIF Quantum, Pleasanton, California). Electron micrographs were recorded with the software SerialEM v3.8.0beta24 on a K2 Summit direct detector (Gatan Inc., GIF Quantum, Pleasanton, California).

## RESULTS

### CF expression and membrane insertion of MS2-L derivatives

CF expression eliminates problems with toxic effects upon overproduction of MS2-L and similar toxins. In addition, the synthesized hydrophobic proteins can already be co-translationally inserted into provided lipid bilayers such as NDs. The combination of CF expression and ND technology thus enables the generation of purified samples of toxins inserted into native-like lipid environments for *in vitro* biochemical analysis. The coding sequences of the full-length toxin MS2-L as well as of a series of five nested deletions of the N-terminal soluble domain starting with deletion of the first 22 amino acids up to position 35 were cloned into pET expression vectors [Supplementary Figure 1]. For MS2-L, a derivative containing a C-terminal GFP fusion was additionally constructed. Furthermore, an N-terminal mScarlet-MS2-L fusion protein was designed. All constructs contained either C-terminal His_10_ or StrepII-tags for purification and immunoblot detection.

The peptides were first CF synthesized in the precipitate forming (P-CF) mode without the supply of hydrophobic agents. All proteins were efficiently expressed, with estimated synthesis yields of between 0.5 and 0.8 mg protein per mL of reaction mixture (RM) as determined by SDS-PAGE analysis [Supplementary Figure 2]. The generated precipitates could be solubilized in the relatively harsh detergent 0.75% 1-myristoyl-2-hydroxy-*sn*-glycero-3-[phospho-*rac*-(1-glycerol)] (LMPG), while milder detergents such as DPC or n-dodecyl-β-D-maltopyranoside (DDM) were ineffective. We therefore proceeded to co-translationally solubilize the synthesized proteins in the presence of various supplied detergents in the detergent-based (D-CF) mode. The peptides were synthesized in the presence of 0.4% of either Brij78, Brij35, or glyco-diosgenin (GDN). The MS2-L derivatives were almost completely soluble in Brij78 and approx. to 70% in Brij35 and to 40% in GDN, respectively. Brij78 was therefore selected for further studies using D-CF synthesized MS2-L derivatives.

In a third approach, MS2-L and MS2-Lp_23_ were synthesized in the presence of supplied preformed nanodiscs (NDs). The NDs were preformed with either DMPC or DMPG *in vitro* and added to the CF expression reactions in concentrations from 5 to 80 µM [Supplementary Figure 3]. After expression, the solubilization of the synthesized MS2-L peptides was monitored via immunoblotting of supernatant and precipitate fractions. At 80 µM ND concentrations, both constructs were finally solubilized to 80%-100%. However, there are apparent differences in the solubilization kinetics of the full-length MS2-L toxin and of the derivative containing a truncated soluble domain. According to the expression rates, the concentrations of the MS2-L peptides in the CF reactions can roughly be estimated to be 60-100 µM. The truncated MS2-Lp_23_ construct achieved complete solubilization at ND concentrations as low as 20 µM, indicating multiple monomer insertions into one ND. In contrast, the full-length toxin MS2-L showed a continuous ND concentration-dependent solubilization with the highest values at approx. 80 µM NDs. This result gives the first evidence of a potential negative effect of the soluble domain on the membrane insertion property of MS2-L. No preference in membrane insertion could be detected for the lipid head group charge [Supplementary Figure 3]. In further tests with 1,2-dioleoyl-sn-glycero-3-phospho-(1’-*rac*-glycerol) (DOPG) and 1-palmitoyl-2-oleoyl-sn-glycero-3-(1’-*rac*-glycerol) (POPG) having increased lipid chain length and lipid flexibility, no difference in membrane insertion, if compared with DMPG NDs, was detected (data not shown).

### DnaJ interaction with MS2-L in NDs and in micelles

The open access of CF reactions allows to synthesize MS2-L in the presence of supplied potentially interacting proteins and to study effects on functional folding, membrane insertion or stability. Former studies suggested an interaction of MS2-L with the chaperone DnaJ^[4]^. In a related system with the phage toxin ΦX174-E, we previously demonstrated that interaction with the chaperone SlyD was necessary to solubilize the nascent toxin inside the cytoplasm and to promote its membrane insertion^[17]^. We therefore analyzed the effects of a potential DnaJ interaction on membrane insertion, assembly and solubility of MS2-L constructs. To have control over the DnaJ concentration in the CF expression reaction, S30 lysates were prepared from the *dnaJ* negative strain BW25113ΔDnaJ^[20]^. The DnaJ protein was furthermore overexpressed in *E. coli* and purified via its C-terminal His_6_-tag as specified in the methods section.

**Figure 1:**
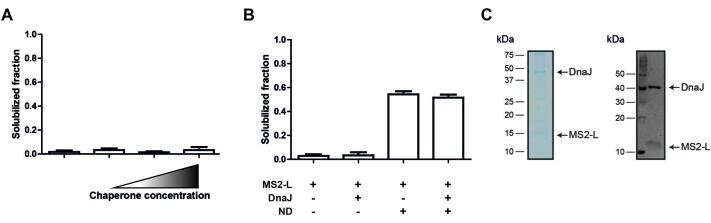
Effect of DnaJ on the co-translational solubilization and membrane insertion of MS2-L. All expressions were carried out in BW25113ΔDnaJ lysate (A) MS2-L was P-CF expressed in the presence of increasing DnaJ concentrations (0, 10, 30, and 100 µM). Solubilization efficiencies were determined by densitometry from immunoblots of pellet and supernatant fractions. Combined signals of pellet and supernatant were normalized to 1. Error bars represent the SEM of *n* = 6 (*n* = 5 for 100 µM DnaJ) biological replicates; (B) MS2-L was either P-CF or L-CF (10 µM DMPG NDs) synthesized in the presence or absence of 100 µM DnaJ. Solubilized MS2-L was quantified as described in (A). Error bars indicate the SEM of *n* = 6 (*n* = 5 for P-CF + 100 µM DnaJ) biological replicates; (C) Complex formation of DnaJ with D-CF synthesized MS2-L. After expression in the presence of Brij78 and 30 µM DnaJ, the toxin was StrepII-purified from the RM. As verified by SDS-PAGE (left panel) and immunoblot (right panel), the chaperone (containing a His_6_-tag) co-purified with MS2-L. D-CF: Detergent-based cell-free expression; DMPG: 1,2-dimyristoyl-sn-glycero-3-phospho-(1’-rac-glycerol); ND: nanodisc; P-CF: precipitate forming cell-free expression; RM: reaction mix.

First, we analyzed whether DnaJ interaction is necessary for the solubilization and membrane insertion of MS2-L. The toxin was synthesized either in the P-CF mode or in the presence of NDs (DMPG) in the BW25113ΔDnaJ lysate in the absence or presence of supplemented purified DnaJ protein [Figure 1A and B]. The solubilization of MS2-L was monitored after CF expression via densitometric analysis of immunoblots of pellet and supernatant fractions. In the P-CF mode, all synthesized MS2-L protein precipitated, independent of the presence of supplied DnaJ [Figure 1A]. Furthermore, the co-translational insertion of MS2-L into provided ND (DMPG) membranes was similarly efficient in the absence or presence of 100 µM DnaJ [Figure 1B].

In the next approach, possible interactions between MS2-L and DnaJ were analyzed *in vitro* in CF reactions. First, purified DnaJ was added to D-CF expression reactions of MS2-L with Brij78 and the toxin was purified after expression via its StrepII-tag. DnaJ was co-purified with MS2-L from the RM, indicating a stable toxin/chaperone complex that is formed in detergent either co-translationally or post-translationally [Figure 1C].

**Figure 2:**
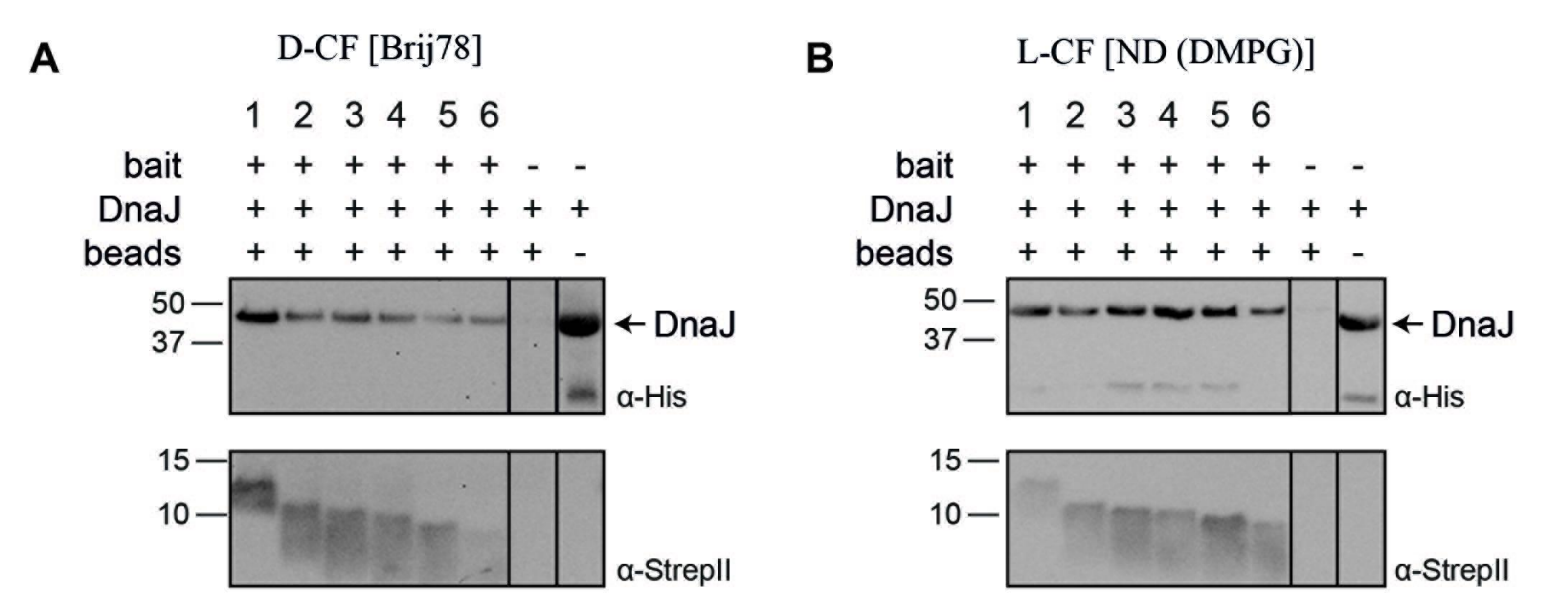
Post-translational interaction of DnaJ with MS2-L derivatives in detergent and in NDs. Toxin expressions were carried out in the BW25113ΔDnaJ lysate. MS2-L derivatives were expressed and solubilized co-translationally in either Brij78 (D-CF) or DMPG NDs (L-CF). Purified toxins were then analyzed in pulldown experiments for interaction with DnaJ. The MS2-L derivatives were immobilized via their C-terminal StrepII-tag and 50 µg purified DnaJ was added as prey. After washing, the formed complexes were eluted in 40 µL of SDS-loading buffer and 15 µL samples were analyzed by SDS-PAGE. Bound DnaJ was detected by immunoblotting with α-His antibodies and immobilized MS2-L derivatives were visualized with α-StrepII antibodies. (1) MS2-L; (2) MS2-Lp_23_; (3) MS2-Lp_26_; (4) MS2-Lp_29_; (5) MS2-Lp_32_; (6) MS2-Lp_35_. (A) D-CF expression in Brij78; (B) L-CF expression in DMPG NDs. The smaller band detected in the DnaJ control is a putative degradation product. D-CF: Detergent-based cell-free expression; DMPG: 1,2-dimyristoyl-sn-glycero-3-phospho-(1’-rac-glycerol); ND: nanodisc; P-CF: precipitate forming cell-free expression.

Furthermore, the post-translational interaction of DnaJ with MS2-L derivatives solubilized in either Brij78 detergent or NDs was analyzed in pulldown assays. Full-length MS2-L as well as constructs containing deletions of the N-terminal soluble domain and resulting in truncations of the first 22 (MS2-Lp_23_), 25 (MS2-Lp_26_), 28 (MS2-Lp_29_), 31 (MS2-Lp_32_) and 34 (MS2-Lp_35_) amino acids were analyzed. The constructs were CF synthesized in BW25113ΔDnaJ lysate and either inserted into NDs (DMPG) or solubilized in Brij78 in the D-CF mode. After expression and subsequent purification, the solubilized MS2-L derivatives were immobilized via the C-terminal StrepII tags on magnetic beads. Purified DnaJ was added as prey and putative complexes were eluted after removal of unbound DnaJ by washing. All MS2-L constructs synthesized in the presence of Brij78 captured DnaJ, as verified by positive pulldown results [Figure 2A]. Some reduced but still detectable DnaJ binding was observed with the shortest construct MS2-Lp_35_. Accordingly, all analyzed MS2-L constructs inserted into NDs (DMPG) did also result in a pulldown of DnaJ, indicating that the relevant binding site remains accessible in the membrane inserted MS2-L and its derivatives [Figure 2B]. The reduced binding of the construct MS2-Lp_35_ to DnaJ furthermore suggests that the binding site is not exclusively located within the soluble domain of the toxin, but may as well cover parts of the trans-membrane domain.

### Formation of oligomeric MS2-L complexes in lipid and detergent environments

**Figure 3:**
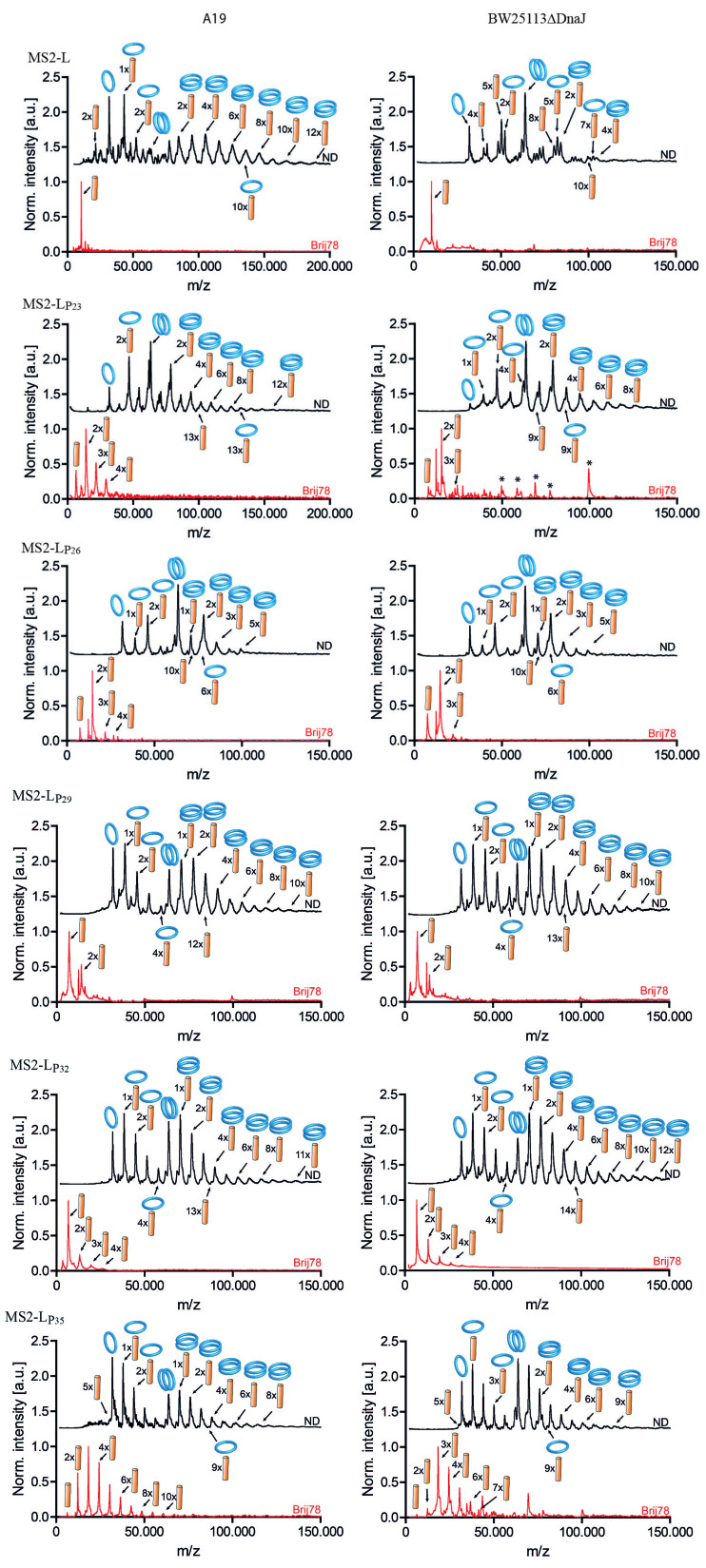
Oligomeric assembly of MS2-L derivatives in NDs and in detergent environments. MS2-L derivatives were CF expressed in the presence of either 60 µM DMPG NDs (black traces) or 0.4% Brij78 (red traces), purified and subjected to LILBID analysis. Samples were synthesized in the standard A19 lysate (left panel) or in BW25113ΔDnaJ lysate (right panel). Pictograms illustrate complexes assigned to the respective peak. For reasons of clarity, not each peak has been assigned a pictogram. Laser intensities were adjusted between 10 and 20 mJ to optimize ion yield while preserving high-order complexes. Asterisks mark prominent peaks representing impurities. The individual MS2-L derivatives are indicated. DMPG: 1,2-dimyristoyl-sn-glycero-3-phospho-(1’-rac-glycerol); LILBID: laser-induced liquid bead ion desorption; ND: nanodisc.

Native LILBID mass spectrometry is an established tool to analyze the oligomeric state of membrane proteins in NDs and other environments^[17,26,28]^. By tuning the intensity level of the laser pulses, the detection of complete or partially dissociated sample complexes dependent on their stability can be achieved. MS2-L samples were first synthesized in the standard S30 lysate either in the presence of 60 µM NDs (DMPG) or in the D-CF mode in the presence of Brij78. In order to reveal the effects of the N-terminal soluble domain on MS2-L oligomerization, full-length MS2-L and the truncated constructs containing deletions of the N-terminal domain were analyzed [Figure 3, left panels]. High oligomeric assemblies containing at least 10 monomers were detected with ND samples containing full-length MS2-L as well as with all five MS2-Lp derivatives [Figure 3, left panel, black trace]. Overall, the stoichiometry of the oligomeric assemblies in lipid environments appears to be similar within all of the six constructs. It is important to note that high oligomeric complexes of the corresponding MS2-L derivative are not just held together by the scaffold proteins, but form stable homomeric complexes. This is visible by the detection of complexes from which the MSP1E3D1 scaffold proteins were stripped off during the LILBID process.

In contrast to the oligomerization in NDs, in detergent, only monomers of full-length MS2-L are detectable [Figure 3, left panel, red trace]. However, lower oligomeric assemblies containing up to four monomers are already formed in detergent by the truncated toxins MS2-Lp_23_, MS2-Lp_26_, MS2-Lp_29_, and MS2-Lp_32_. N-terminal deletion of further three amino acids in MS2-Lp_35_ completely restored the high oligomerization observed in NDs and assemblies containing up to 10 monomers could be detected.

As bacterial cell lysis by MS2-L may be modulated by DnaJ, we next analyzed whether the chaperone has any effect on the oligomerization state of the toxin. MS2-L and the truncated MS2-Lp derivatives were CF synthesized in BW25113ΔDnaJ lysate in the presence of NDs (DMPG) or Brij78 micelles, and the samples prepared as above were analyzed by LILBID-MS [Figure 3, right panels]. The spectra of oligomeric complexes of all samples prepared in BW25113ΔDnaJ lysate were similar to those prepared in the standard S30 lysate. This indicates that interaction with DnaJ has most likely no direct impact on the homomeric oligomerization of MS2-L, neither in detergent nor in ND (DMPG) membranes.

### *In vivo* cluster and pore formation of MS2-L derivatives

Complementary to the *in vitro* studies, the *in vivo* effect of full-length MS2-L derivatives in the *E. coli* strain Lemo21 (DE3) was analyzed. Plasmids encoding for the constructs MS2-L, MS2-L-GFP and mScarlet-MS2-L were transformed into Lemo21 (DE3). Growth curves of the cells were recorded after induction of toxin expression by IPTG to determine the cell lysis behavior [Supplementary Figure 4]. All MS2-L constructs showed similar toxic effects and inhibited further growth of the cells shortly after induction.

**Figure 4:**
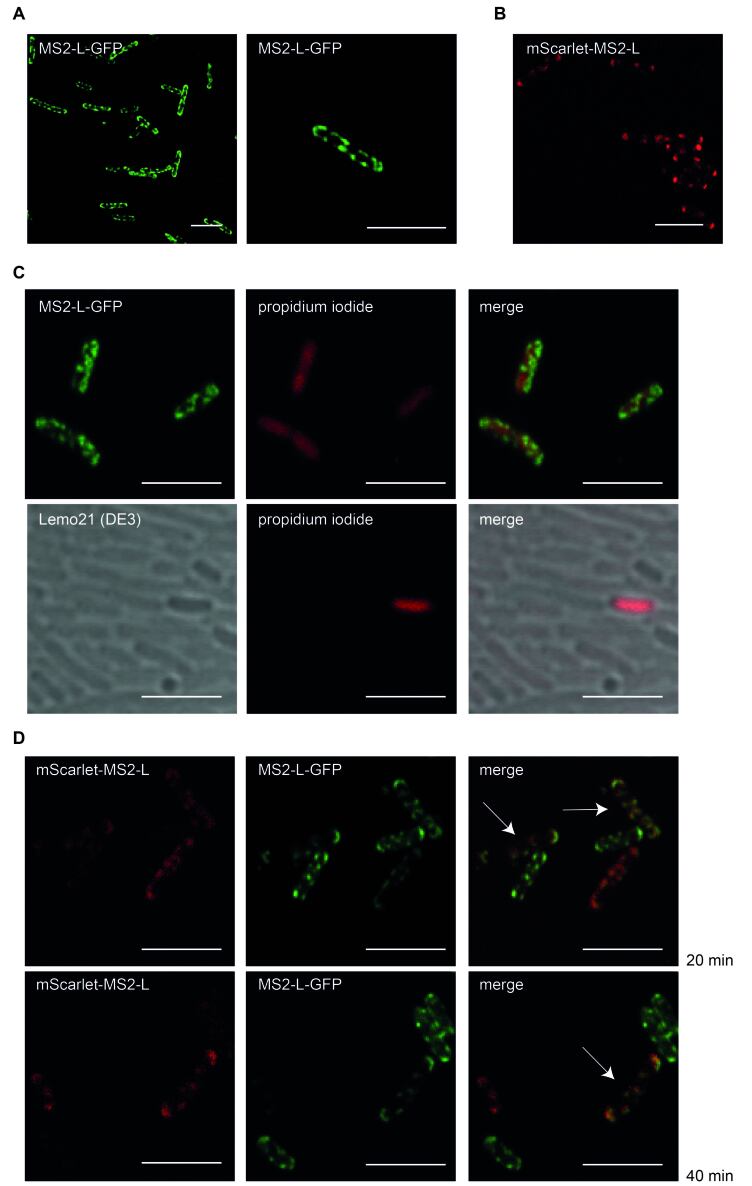
Cluster formation of full-length MS2-L derivatives in *E. coli* Lemo21 (DE3) bacterial cells. Toxin expression was induced at OD_600_ = 0.3 with 1 mM IPTG and samples were taken at defined time points after induction. All images were acquired with an LSM700 confocal microscope with a 63× objective (scale bar = 5 µm). (A) Cells synthesizing MS2-L-GFP 45 min after induction; (B) Cells synthesizing mScarlet-MS2-L 40 min after induction; (C) Propidium iodide staining of cells synthesizing MS2-L-GFP 45 min after induction and of Lemo21 (DE3) control cells. The vast majority of the control cells remain unstained; (D) Co-expression of MS2-L-GFP and mScarlet-MS2-L, 20 and 40 min after induction. GFP: Green fluorescent protein; IPTG: isopropyl-β-D-thiogalactopyranoside.

**Figure 5:**
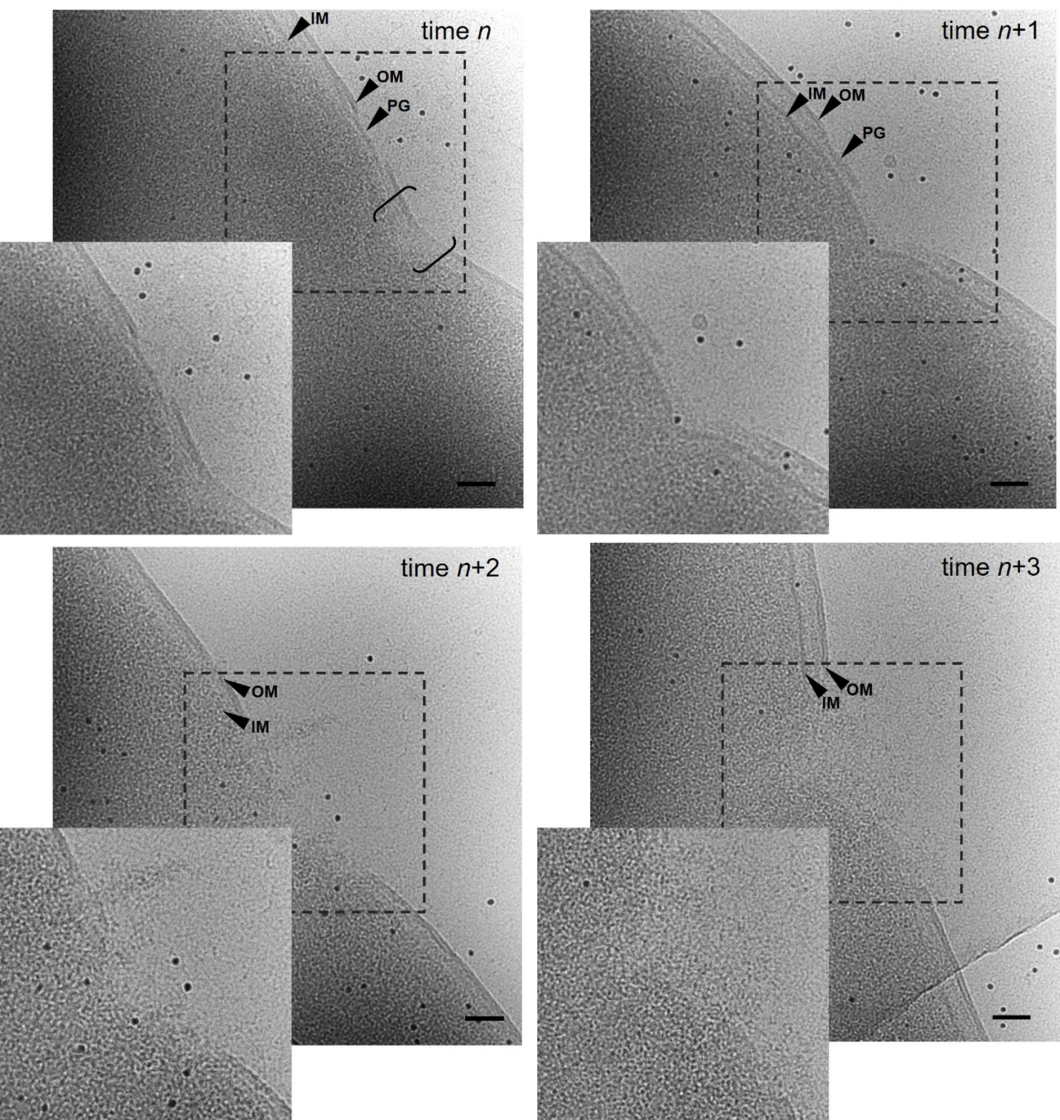
Lesion formation in *E. coli* Lemo21 (DE3) cells synthesizing MS2-L. Toxin expression was induced at OD_600_ = 0.27 with 1 mM IPTG and samples were taken 60 min after induction and immediately vitrified. Electron microcopy images were acquired with a Titan Krios and a K2 Summit direct detector. Different stages of lesion formation are shown in a proposed sequential order. Data were acquired at a magnification of 81,000x at a defocus of -50 µm and with 30-40 e-/Å total dose (scale bar = 0.05 µM). OM (outer membrane), IM (inner membrane) and PG (peptidoglycan) are indicated by arrows. The fission zone is indicated by brackets. IPTG:Isopropyl-β-D-thiogalactopyranoside.

Samples of cells expressing the full-length derivatives MS2-L, MS2-L-GFP and mScarlet-MS2-L were taken at several time points after induction and analyzed by fluorescence and electron microscopy [Figures 4 and 5]. The cells showed numerous large fluorescent clusters located in the periphery of the cells, indicating a membrane integration [Figure 4A and B]. The clusters were detectable 10 minutes after induction and became more prominent with extended induction times. Similar clusters were observed after the expression of the constructs in T7 Express cells (data not shown). PI staining revealed that the cluster formation of MS2-L in bacterial cells was accompanied by membrane permeabilization, probably due to pore formation. The vast majority of control cells did not take up the dye [Figure 4C]. The pore formation could be verified with electron microscope images of vitrified bacterial cells. Pores at different formation stages were detected in most cells in samples taken 60 min after induction of the full-length toxin MS2-L [Figure 5]. Apparently, the outer membrane is disrupted first, followed by disintegration of the underlying peptidoglycan layer. In the next step, the inner membrane is disrupted, causing excessive leakage of cytoplasmic content. No fusion of the outer and inner membranes at the periphery of the pores could be observed.

The fluorescent clusters could represent large assemblies of the toxins in the cell membranes and would thus support our observation of oligomer formation in ND membranes *in vitro*. To provide further evidence of MS2-L oligomerization in the bacterial membrane, we analyzed for co-localization of MS2-L-GFP and mScarlet-MS2-L in Lemo21 (DE3) cells. Templates for MS2-L-GFP and mScarlet-MS2-L were co-transformed into Lemo21 (DE3). The transformed cells were double-selected on ampicillin and kanamycin and then inoculated in liquid medium containing both antibiotics. Samples were taken at different time points (20 and 40 min) after IPTG induction and analyzed for GFP and mScarlet fluorescence [Figure 4D]. Approx 20% of the cells showed fluorescence of both monitors and the corresponding clusters perfectly matched in overlays.

## DISCUSSION

The role of MS2-L in bacterial cell lysis remains a matter of debate. Electron micrographs revealed large cell wall lesions associated with the efflux of cytoplasmic content in MS2-L expressing *E. coli* cells^[2]^. In combination with the observed localization of MS2-L at membrane adhesion sites, lesion formation by direct involvement of MS2-L was suspected^[5]^. Expression of the C-terminal MS2-L transmembrane domain is sufficient to induce bacterial lysis and leakage in liposomes as well as in *E. coli* membrane vesicles. This was shown with synthetic peptides covering the C-terminal 25 amino acids of MS2-L^[3,6]^. A second and potentially simultaneous mechanism is the activation of the bacterial autolytic system by MS2-L^[1,29]^. Lesion formation would then be supported and associated by the activity of murein hydrolases. By combining both mechanisms, a two-step process was suggested where membrane interaction of MS2-L first results in membrane depolarization. This triggers the activation of autolytic enzymes, which then locally generate large holes in the murein sacculus^[6]^. The biological relevance for such a dual action of MS2-L could be a more efficient release of the approx. 28 nm MS2 phage through the relatively tight murein-sacculus into the environment.

By our combined approach including CF expression, ND technology and native LILBID MS, we could show for the first time the high-order self-assembly of MS2-L monomers triggered by its transmembrane domain. In combination with the fact that the C-terminal domain is also essential for lysis, this could support the possibility of the participation of MS2-L higher-order assemblies in the disintegration of the bacterial cell envelope. The assembly of MS2-L resembles previous data obtained with the related lysis toxin ΦX174-E. Similar to MS2-L, in ΦX174-E, the lysis activity is confined to the transmembrane domain, which additionally promotes the oligomerization process^[2,17,30-32]^.

The MS2-L soluble domain is dispensable for cell lysis and no function could be attributed so far. It is encoded by overlapping sequences with the phage coat protein, leading to speculations that it might be rather a byproduct of the control mechanisms in the MS2 phage expression process^[3,33,34]^. As some first effects of the MS2 soluble domain, we show that its presence in MS2-L impairs the oligomeric assembly in detergent environments. Furthermore, the insertion kinetics of MS2-L into a ND membrane was different if compared with the construct MS2-Lp_23_ truncated in the soluble domain. These effects hint at a potential regulatory function of the MS2-L N-terminal domain, similar to that observed with the soluble domain of the toxin ΦX174-E^[17]^. Here, a conformational lock formed by the intramolecular interaction of soluble and transmembrane domains prevents membrane insertion and oligomerization. This intramolecular lock is released by interaction with the chaperone SlyD. The biological relevance of this mechanism could be a quorum sensing mechanism that contributes to determining optimal time points for phage release^[8]^. However, despite these coincidences, the effect of the soluble domain in the MS2 system seems to be different. Its inhibitory function on MS2-L oligomerization was not observed in membrane environments of NDs and membrane insertion was retarded but not completely prevented as in the ΦX174-E system. However, our results were obtained *in vitro* with plain lipid bilayers devoid of any other proteins. The insertion and oligomerization of MS2-L in crowded native bacterial membranes will be a different situation and modulatory effects of the soluble domain could then become more important.

A previously identified interaction partner for MS2-L is the chaperone DnaJ^[4]^. We confirmed previous *in vivo* studies by demonstrating strong binding of DnaJ to the MS2-L soluble domain in the combined CF/ND system. We further showed that DnaJ also binds post-translationally to MS2-L and binding extends at least to amino acid position 35, being very close to the proposed transmembrane domain. This is in clear contrast to the ΦX174-E interaction with the chaperone SlyD that only happens co-translationally^[17]^. Moreover, DnaJ is also not able to keep MS2-L in a soluble, membrane insertion competent complex as observed with the ΦX174-E/SlyD interaction. The DnaJ binding site of MS2-L remains accessible after its membrane insertion and strong binding was monitored in truncations of the soluble domain up to amino acid position 32. The reduced but still detectable DnaJ binding after further deletions up to amino acid position 35 indicates that the binding site extends close to the proposed transmembrane domain. Approx. 25 amino acids are sufficient to span a membrane and the 41 amino acid construct MS2-Lp_35_ could still contain protruding parts sufficient to interact with DnaJ^[3,35]^. The post-translational interaction could indicate a function of DnaJ in coordination with the soluble domain to increase the efficiency of MS2-L membrane insertion and oligomerization in more complex cell membranes. This would be in agreement with the observed faster cell lysis of N-terminal truncated MS2-L derivatives *in vivo*^[4]^. However, these effects appear to be rather modulatory in view of the observation that DnaJ is not essential for MS2-mediated bacterial lysis^[4]^. Despite its different action, DnaJ might thus have a similar but less restrictive function as SlyD in the timing of bacterial lysis and MS2 phage release.

MS2-L localizes in the bacterial cytoplasmic membrane and, to a lesser extent, in the outer membrane^[1,5]^. Furthermore, a significant increase in adhesion sites upon MS2-L lysis was noticed and approx. 30% of MS2-L finally localized in clusters associated with newly formed adhesion sites^[5]^. MS2-L lysis further requires a certain spacing of inner and outer membranes, indicating the formation of rather defined high-order structures^[36]^. By LSM microscopy, we visualized cluster formation of overexpressed MS2-L derivatives in the periphery of bacterial cells. The cluster formation, in contrast to a random distribution, agrees with the *in vitro* data obtained with NDs and supports an oligomeric assembly *in vivo* as well. Furthermore, global cellular influx of PI was only detected in cells containing MS2-L clusters, indicating that the cells were lysed and at least some of the clusters could be associated with lesions in the cell wall. In ND membranes, we could detect up to dodecameric complexes of MS2-L. However, complex disintegration by the laser power upon LILBID-MS analysis might restrict the detection of higher oligomers. Furthermore, it is not clear yet whether a distinct MS2-L stoichiometry in complex assembly or simply large cluster formation is necessary to cause membrane and cell wall disruption.

The cryo-electron microscopy studies provided initial insights into the likely first steps in lesion initiation. Previous data obtained by transmission electron microscopy revealed the local disruption of large areas of the bacterial cell envelope by expression of MS2-L derivatives^[2]^. The high release of periplasmic marker proteins further indicated that the inner and outer membranes did not fuse during lesion formation. Its initiation was proposed by permeabilization of the inner membrane and followed by a burst of the outer membrane due to over-expansion caused by leakage of cytoplasmic content^[2]^. The analysis of MS2-L directed cell wall disruption by high-resolution cryo-electron microscopy partially agrees with that finding as, indeed, no connection between the inner and outer membranes can be observed. However, lesion formation appears to be initiated by the disintegration of small areas of the outer membrane, while the inner membrane in those areas remains structured. Nevertheless, molecular changes of the inner membrane in the lesion areas resulting in increased permeability and release of cytoplasmic content can still not be ruled out. Lesion formation proceeds by disintegration of larger areas of the outer membrane and the underlying peptidoglycan layer followed by disintegration of the inner membrane. The detected lesions localize predominantly in the close vicinity of cell fission zones. Whether MS2-L oligomers directly or indirectly cause local permeabilization of the inner membrane or whether they are even localized in the outer membrane can currently only be speculated.

The focus of this study was to further pave the way for directed pharmacological applications of MS2-L and related toxins. It needs to be considered that the presented results are obtained either with purified protein or by overexpressing toxin derivatives in cellular context. Whether similar mechanisms occur during coordinated phage infection of bacterial cells still needs to be demonstrated. Despite the similar two-domain structure, no explicit amino acid conservation within the two peptides MS2-L and ΦX174-E is detectable. Furthermore, the inhibition of a distinct target such as MraY by ΦX174-E has not been detected for MS2-L yet. Inhibition of cell-wall biosynthesis by MS2-L is supposed to be rather unlikely, as no accumulation of cell-wall precursor has been observed^[29]^. Accordingly, we could not detect any interaction of MS2-L in NDs with the lipid I precursor forming enzymes MurA-F in pulldown assays (data not shown). However, inhibition of later steps in cell wall formation and interaction of MS2-L with a yet non-identified target can still not be ruled out. DnaJ might also play a role in keeping the MS2-L conformation competent for such an interaction^[4]^. Based on the demonstrated ability of MS2-L and ΦX174-E to oligomerize, a likely function could be their participation in the formation of membrane disintegrating or penetrating pores as at least an important asset of cell lysis. A pore-forming ability is common to a large variety of naturally occurring peptide antibiotics and a variety of basic structures is possible^[37-40]^. The oligomeric interface of MS2-L and ΦX174-E is located within their transmembrane domains. Both domains share a common hydrophobic leucine-rich region essential for lytic activity^[41]^. The high-order assembly of MS2-L and ΦX174-E *in vitro* might be a paradigm for a large number of related small phage toxins and could become an important feature in future applications.

## DECLARATIONS

### Copyright

© The Author(s) 2023.
