# Supplementary material for: In vitro characterization of the phage lysis protein MS2-L
Source: Microbiome Res Rep. 2023 Jul 20;2(4):28. doi: 10.20517/mrr.2023.28 (PMC10688784; doi:10.20517/mrr.2023.28)
Supplement: Supplementary file 1 [file mrr-2-4-28-SupplementaryMaterials.pdf]

Supplementary Materials

In vitro characterization of the phage lysis protein MS2-L

Julija Mezhyrova[1], Janosch Martin[2], Clara Börnsen[3], Volker Dötsch[1], Achilleas Stefanos Frangakis[3], Nina Morgner[2], Frank Bernhard[1*]

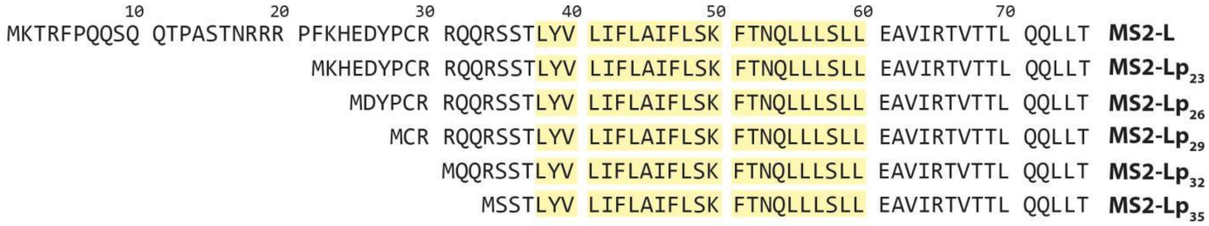

Supplementary Figure 1. Amino acid sequences of MS2-L derivatives. Full-length MS2-L was gradually truncated at the N-terminally located soluble domain. The transmembrane region, predicted by the interactive protein feature visualization tool PROTTER (ETH Zürich), is highlighted in yellow. All constructs were synthesized with a C-terminal StrepII-tag separated by a short linker (MS2-L-GTGG-WSHPQFEK).

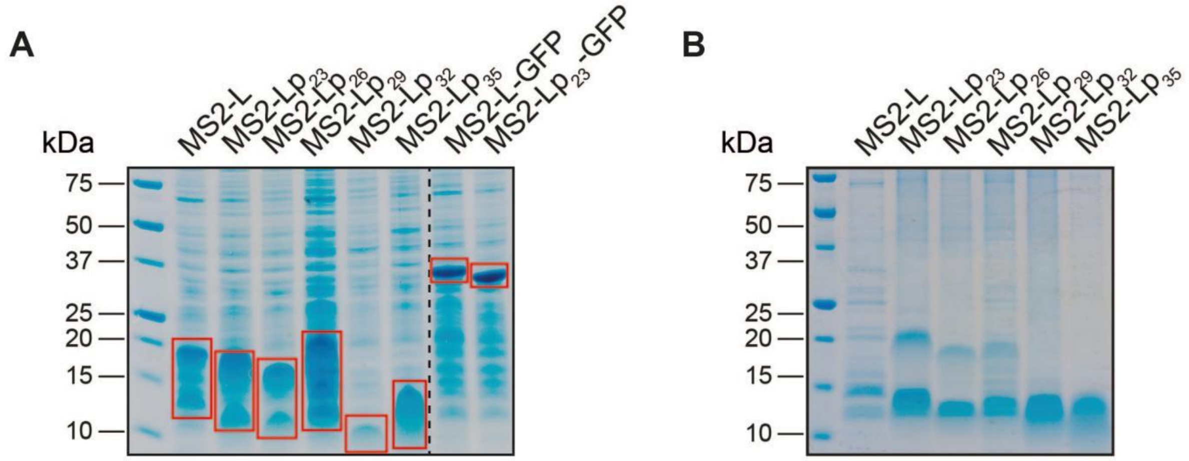

Supplementary Figure 2. CF expression and purification of detergent solubilized MS2-L derivatives. All proteins were CF synthesized at a previously determined Mg^{2+} optimum of 20 mM. (A) Exemplary P-CF expression. After harvesting, the pellets were suspended in S30 buffer C in the respective RM volume and 1.5 µL of the sample was analyzed on a discontinuous 4%-11 % Tris-Tricine SDS-PAGE. Overexpression bands are framed by red boxes. Other bands appearing on the SDS-PAGE correspond to co-precipitated proteins from the S30 lysate; (B) Exemplary StrepII-purified MS2-L derivatives after D-CF expression with detergent Brij78.

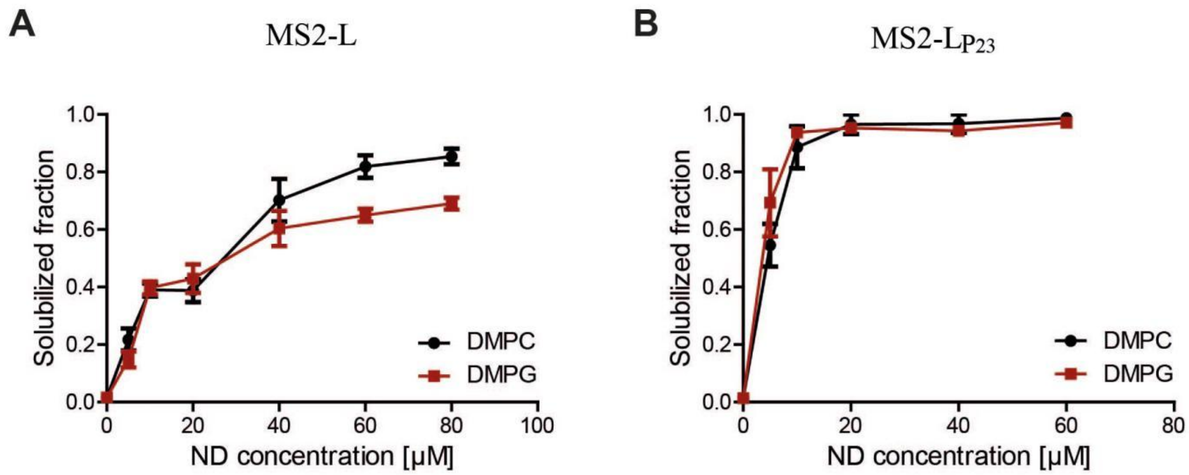

Supplementary Figure 3. Co-translational insertion of MS2-L and MS2-Lp23 into NDs. CF reactions were supplemented with increasing concentrations of preformed NDs. After expression, the samples were centrifuged and pellets were suspended in S30 buffer C in the respective RM volume. 3 µL of pellet and supernatant fractions were separated by SDS-PAGE and the amount of peptide in both fractions was determined by densitometry of immunoblots. The combined signal was normalized to 1 and the relative amounts of solubilized peptide/ND complexes were calculated. Error bars represent the SEM. (A) MS2-L; DMPC (n = 6 for 5, 10, 20, 40, 60 µM; n = 4 for 0 µM; n = 3 for 80 µM), DMPG (n = 4 for 0, 20, 40, 60, 80 µM; n = 3 for 5, 10 µM); (B) MS2-Lp23; DMPC (n = 3 for all concentrations), DMPG (n = 3 for 0 µM, n = 4 for 10 µM, n = 5 for 20, 40, 60 µM and n = 6 for 5 µM).

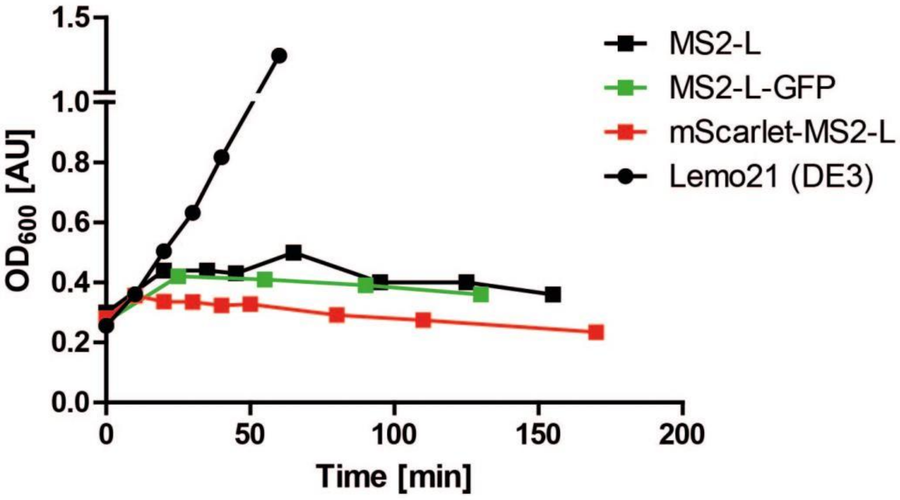

Supplementary Figure 4. Representative growth curves of Lemo21 (DE3) cells expressing MS2-L derivatives. Liquid cultures were inoculated with freshly plated transformed cells and induced with 1 mM IPTG at early log phase (t = 0). Cell growth was monitored over time at the indicated time points after induction.
